# Navigating the Gap: An Examination of Funder Rhetoric and Reality Regarding Community‐Research Partnerships

**DOI:** 10.1111/dar.70084

**Published:** 2025-12-22

**Authors:** Danielle M. Russell, Ele Morrison, Jason Grebely, Carla Treloar, John Gobeil, Jess Doumany, M. J. Stowe, Paul Dietze, Kate Seear, Natalie Taylor, Alison Ritter

**Affiliations:** ^1^ The Kirby Institute University of New South Wales Sydney Australia; ^2^ Australian Injecting and Illicit Drug Users League Canberra Australia; ^3^ Centre for Social Research in Health University of New South Wales Sydney Australia; ^4^ Burnet Institute Melbourne Australia; ^5^ National Drug Research Institute Curtin University Melbourne Australia; ^6^ Deakin University Melbourne Australia; ^7^ Australian Research Centre in Sex, Health and Society La Trobe University Melbourne Australia; ^8^ Implementation to Impact, The School of Population Health University of New South Wales Sydney Australia; ^9^ Drug Policy Modelling Program University of New South Wales Sydney Australia

**Keywords:** community‐based participatory research, consumer participation, health policy, research funding, substance use disorders

## Abstract

Partnerships between researchers and consumers—specifically, people who use drugs in the context of our research co‐production—can significantly enhance the relevance and impact of research. Funders increasingly emphasise the value of community‐researcher collaborations, yet translating this commitment into effective practice remains challenging. This commentary draws from our experience in developing an application for a collaborative grant with the National Health and Medical Research Council (NHMRC) in Australia, focusing on issues that impact people who use drugs. We reflect on four key insights that emerged from the grant‐writing process: (i) expectations and resources, highlighting the gap between the expectations of funding bodies, the way resourcing is used in academic contexts and the resources required for meaningful community involvement; (ii) grant success metrics, examining the disconnect between funding criteria and the realities of co‐production; (iii) representation, addressing the challenges of ensuring equitable and authentic consumer participation; and (iv) sharing power, discussing how power dynamics between researchers and community members influence the research process. In conclusion, we propose that researchers must work with consumers to foster more equitable working relationships and ensure there are adequate resources to support meaningful collaborations. Funding bodies, in turn, need to reconsider success metrics and ensure that their processes are mindful of the practical requirements that are necessary to facilitate genuine, equitable partnerships between researchers and consumers.

## Key Point Summary

- *Discrepancy Between Policy and Practice*: While institutions like the National Health and Medical Research Council and others, often emphasise consumer and community involvement, their grant processes create barriers that limit the meaningful participation of consumers.
- *Structural and Financial Barriers*: Researchers often lack the necessary time and resources to engage consumers early in the research process, and consumers may generally have even less support to facilitate engagement, making equitable collaboration difficult.
- *Grant Success Metrics Favour Academics Over Lived‐Living Experience*: Current funding criteria prioritise traditional academic achievements, undervaluing the expertise of community members with lived‐living experience and expertise.
- *Challenges in Representation and Power Sharing*: Tensions exist between research institutes, researchers, consumers and consumer organisations in sharing the power that comes with resourcing and decision‐making. There are also challenges around defining ‘community’ and ensuring that those consumers involved in research have the necessary resourcing, capacity and connections to ensure there is genuine representation from the broader population of consumers.
- *Need for Systemic Change in Research Funding*: Funders should revise grant application processes to better support co‐produced research and to demonstrate that their actions are aligned with statements that have been made regarding lived‐living experience as a valuable form of expertise.

## 1 Introduction

Partnerships between researchers and people who are directly impacted by research can greatly improve the quality and utility of research outputs and interventions [1]. The potential benefits of co‐produced research extend beyond increased impact, providing reciprocal learning and capacity‐building opportunities to both researchers and community members [2, 3]. Actively involving people who use drugs in the design, implementation and evaluation of research helps ensure that research is ethical, culturally appropriate, contextually relevant and responsive to consumer and community needs [4, 5, 6, 7].

Globally, research that is conducted as a collaborative effort between academics and community members has become increasingly valued, particularly by funders. In Canada, for example, many funding agencies require community members to be co‐applicants, recognising the importance of these collaborations [8]. In the United States, the Director of the National Institute on Drug Abuse (NIDA), Nora Volkow has also recognised the importance of consumer involvement in research, highlighting the utility of collaborative research to address complex public health challenges, particularly in underserved populations [9]. The National Institute for Health and Care Research in the United Kingdom outlines seven principles that should help to guide community engagement and involvement in global health research [10]. Similarly, in Australia, organisations like the National Health and Medical Research Council (NHMRC) have endorsed the value of collaborative involvement with consumers and communities in shaping research priorities and outcomes [11]. While funding agencies increasingly require community members as co‐applicants, there is a lack of well‐defined processes that allow for genuine participation. Despite the call for inclusivity and increased community engagement in research from some funders, it is often unclear what steps researchers have taken to engage the community, or even if the community has been engaged in the research at all [12].

In this commentary, we reflect on our own experiences developing an NHMRC research grant application with the intention that this project would meaningfully engage and be co‐led by consumers through a truly equitable partnership between researchers and community‐led drug user organisations (DUO). The NHMRC is one of the largest government funding organisations for health research in Australia. We note that the NHMRC defines the term ‘consumer’ as ‘patients and potential patients, carers and people who use health care services’ [11]. The way we are using the term ‘consumer’ is intended to refer to the people who would be most directly affected by the research, and who will live with the research impacts. In our case, this is specific to people who use drugs.

Our engagement with the community is through representation—people within academia who use drugs and through community‐led organisations that are led by and for people who use drugs with the aim of advancing dignity, health, equity and human rights of their communities (such as AIVL—the Australian Injecting and Illicit Drug Users League, the Australian peer‐led peak organisation community‐controlled by people who use drugs). Our team has coalesced around a shared understanding that there is a critical need to improve how researchers and consumers work together to define and solve problems. This awareness has helped us to be more intentional and reflective about our process of engagement and partnership. Our efforts to collaborate equitably have provided a significant opportunity to consider the ways in which a funding body (in this case the NHMRC) constitutes policies and procedures and can facilitate and enable (or create barriers to) meaningful involvement of consumers. We briefly describe the current NHMRC context before then reflecting on our experiences. Our hope is that this commentary provides useful reflections for researchers and people who use drugs (and other communities/consumers) who are committed to improving how they work together in establishing meaningful research partnerships. We also hope to inform research institutions and funding bodies about potential areas for improving and increasing the viability and feasibility of meaningful consumer involvement in research.

## 2 NHMRC and Consumer and Community Involvement

Consumer and community involvement appears throughout NHMRC policies and procedures; they are replete with references to meaningful engagement and active involvement of consumers and the community in all aspects of health research. We are aware that at the time of writing this, the NHMRC is reviewing the current overarching statement ‘Consumer and community involvement in health and medical research’ [11]. This document outlines the vision (‘working in partnership’), the benefits, and the differing levels of involvement of consumers in health and medical research. This overarching statement also includes a section ‘putting the statement into practice’ which includes attention to procedures and policies at research institutions, capacity building and resourcing. In line with the *Statement on Consumer and Community Involvement in Health and Medical Research*, NHMRC policies support consumer engagement in the peer review process, including the participation of consumers as full scoring members in targeted research funding rounds. The NHMRC also has a Consumer and Community Advisory Group [13], published a code of conduct for researchers [14], which covers consumer and community involvement, and a series of toolkits and resources for researchers [15] that promote and encourage the equitable inclusion and involvement of consumer and community members across the breadth of the research process.

There is little doubt that the NHMRC demonstrates a high‐level commitment to meaningful involvement of consumers in all aspects of health and medical research and has made available significant resources to its constituents, ostensibly to give effect to this commitment. As a group of researchers and community members who are committed to the ethical imperative of community partnerships in research, we have found the process of developing an application for funding with the NHMRC to be at odds with much of the sentiment documented in these publications about consumer and community involvement in research. The indicators of commitment to support meaningful consumer engagement that can be found across NHMRC websites and resources are suddenly and noticeably absent from much of the machinery of the grant application process.

## 3 Key Differences: From Rhetoric to Reality

Our experience developing a grant application with a diverse, multidisciplinary team of researchers and community members has provided an opportunity to reflect on the differences between the rhetoric surrounding community research partnerships and the reality of their implementation. We have structured our reflections around four key insights: expectations and resources; grant success metrics; representation; and sharing power.

### 3.1 Expectations and Resources

The NHMRC states that consumers should be involved in ‘all stages of the research cycle’, including the earliest phases of conceptualising ideas and setting research priorities [11]. This necessarily includes initial thinking about research agendas for grant proposals. In its *Measuring alignment* toolkit for researchers [15], the NHMRC clearly expects alignment between research agendas and consumer or community expectations. It explicitly asks researchers to consider: ‘Does the research project involve consumers and community members in the research concept/hypothesis/question development?’

In our approach, engagement between researchers and community members (or consumers as defined by NHMRC) began early at the conception of the idea. The lead researcher first contacted representatives from AIVL, the national peer‐led peak organisation community‐controlled by people who use drugs and the Canberra Alliance for Harm Minimisation and Advocacy, one of the state/territory‐based DUOs to have an initial planning meeting to discuss an approach. One of the outcomes from this was to consider a research partnership between community leaders and other peer‐led state/territory drug user organisations and university‐based researchers (including researchers who have living experience with drugs). At one of these early meetings, we discussed the characteristics of the proposed grant scheme and developed a strategy for how we would elicit feedback about critical research priorities from the broader community of people who use drugs. A series of subsequent workshops and meetings were held across 2023 and 2024 to discuss research priorities, identify the proposed investigators for the grant (aligned with the topic and discipline expertise for the proposed priorities identified), establish a scientific plan and propose potential capacity building and knowledge translation activities.

The individuals from the community organisation and the researchers involved in these early discussions, including the lead researcher, had worked together previously. There were existing foundations built on years of partnerships in research, project work, committees and other collaborations. The trust between the community members involved, the lead researcher and the other researchers, some of whom identify as people who use drugs, streamlined project initiation and conserved time and resources. Financial resources to support meaningful engagement are often constrained in universities [16, 17]. In our case the lead researcher was able to contribute funds to support the engagement process (including catering, travel, accommodation costs associated with meetings and salary contributions to the community organisation). Community organisations including AIVL are also under‐resourced, and funding is tied to specific activities and programs. Many community organisations in Australia take on additional unfunded work, particularly in the research space. It is common to provide significant time participating in research committees and advisory boards, giving advice and feedback on research, acting as research participants, promoting research to community members and even acting as interviewers on research projects, often without funding or with small contributions. Participation in the early stages of a grant proposal is far less common. Community organisations provide their time and expertise without any or adequate funding for research proposal development because they believe the outcomes of their participation or the research itself will benefit their organisations, or their community. Additional time where the outcome is not assured, especially given the low rate of success for grant proposals, can be difficult to justify.

Community organisations are already overextended, often working beyond their capacity without adequate recognition or compensation. Meaningfully engaging a broader group of community members who use drugs—particularly those not formally employed within community organisations—requires substantial time and resources. This includes efforts to recruit participants, build trust and provide the financial and structural support necessary to ensure equitable and meaningful participation. Although these considerations apply to all such groups, individuals who use drugs additionally experience stigma and criminalisation and other structural violence that may disrupt or restrict their capacity to safely partner with researchers. Adding to this challenge, many researchers lack the financial support necessary to dedicate time to building their own knowledge base, establishing the trusting relationships required for effective collaboration with communities and providing the resources needed to ensure that all participants can engage meaningfully as full research partners from the beginning of research proposals.

It is also important to acknowledge that some researchers may lack substantive, applicable knowledge about the lives and experiences of people who use drugs. This knowledge gap can contribute to the perpetuation of objectifying, condescending, or stigmatising attitudes, which in turn may alienate community members and undermine efforts to establish meaningful and collaborative partnerships. Individuals who work at drug user organisations are not merely passive recipients of overtures from researchers; they are more likely to partner with researchers who have put time and effort into developing respectful relationships built on shared goals of working towards positive change for the community.

Meaningfully including people who are not immersed in academic language and culture [18] as equal members of the research team takes commitment and time. We experienced many examples where language that was familiar to academic researchers was unfamiliar to the community members. One example was the language of ‘Chief Investigator’ and ‘Associate Investigator’—these labels and associated roles are used by funders and researchers, but are not used by community researchers. Even amongst academic researchers, understandings of key terms and role expectations are neither fixed nor universally shared, often varying across projects. Communication preferences and comprehension also differ. For example, presenting the meeting minutes as visual representations (using CANVA) illustrated how the diversity of the team, especially the presence of people who use drugs, influenced communication choices.

The NHMRC has clearly documented its recognition that consumer engagement requires resources. For example, page 12 of the Statement notes: ‘researchers should have planned budgeted strategies to support, implement and acknowledge appropriate consumer and community involvement in the research process’ [11], yet funding is usually only available after the grant is awarded. Implementing this imperative to include community research partners at the start of the research process was only possible for our team because of the commitment of the community organisations and the willingness of the researchers to invest in the partnership.

### 3.2 Grant Success Metrics

The team was eager to prepare a highly competitive grant application. A review of the assessment criteria and score descriptors used to assess the grant that our team was considering reveals that the rhetoric about and emphasis on the value of consumer and community inclusion in research is largely absent from the NHMRC document outlining how applications are scored and assessed. A successful application would be reliant on a highly competitive track record, which significantly impacted our considerations of who would be listed as a Chief Investigator. Our commitment to power sharing (see below) and living the principles of partnership meant that the team would be including community researchers at all stages and levels, despite some having no or scarce academic track record. However, all parties were required to discuss whether having equal numbers of consumers and researchers in each role would compromise the success of the proposal, a dialogue that had the potential to damage trust between the different parties, as well as exacerbate perceptions of stigma. The necessary considerations regarding how many academic researchers and how many community researchers would be listed as investigators, and how these choices would impact the likelihood of grant funding were important and sensitive discussions among the team members.

The contrast between what has been published in the NHMRC documents and how grant applicants are evaluated was further emphasised to the team when we began to prepare track records for community members to participate as equal members of the research team, as Chief Investigators (CI). Recognition of the expertise of CIs is entirely related to the number of and impact of academic publications. Because the grant had no metrics that would fairly assess the community members we wanted to include as investigators, and they would be assessed against a metric clearly developed for professional academic researchers, our team believed that our efforts to ensure equitable partnership with and the early involvement of community members in the research process would likely reduce our score.

In stark contrast to our experience, the NHMRC Statement on Consumer and Community Involvement in Health and Medical Research [11] makes explicit mention of engaging consumers as Chief Investigators. In the section identifying levels of consumer involvement, the third dot point is ‘as a Chief Investigator on a research project or grant application’ (page 10). However, even the fields that must be completed in the CI track record are quite obviously centred on evaluating individuals who have built their careers as formal academics or researchers and do not recognise the expertise of community members. This narrow vision of the potential impact of research is exemplified by the requirement of an account on ‘Sapphire’ (the NHMRC's grant application and management system) and the inclusion of scholarly publications as the key mechanism to demonstrate impact. This interface is not built to be consumer‐friendly. As asked by one of our community partners ‘is it intentionally alienating, do you think?’ Because the language currently used could not be easily understood by anyone except academic researchers, some people may have the impression that it is deliberately meant to signal belonging or not belonging to an insider group (academics).

The NHMRC does not appear to have provision for acknowledging the expertise of community members without the inclusion of academic papers and research impact. The Canadian Institutes of Health Research can be highlighted as an excellent example of a funding institution taking action to ensure that people from non‐academic backgrounds can be included as equal members of the research team. Applicants who are community‐based can submit a ‘Community CV’ that highlights a broader range of experience, expertise and contributions to knowledge generation [8].

### 3.3 Representation

Representation and identity, including who stands for whom, is highly complex [19, 20, 21]. The labels that are ascribed to ‘consumer’ and ‘community’ matter. DUOs are often referred to as ‘community’ as their membership is constituted of community members who identify as people who use drugs, and the objectives of those organisations are to support and represent people who use drugs. However, for some people who use drugs who are not familiar with these organisations, it is unlikely they would view or refer to drug user organisations as their community. The community of people who use drugs is diverse and constantly growing and changing. Identity is fluid and drug use itself evolves for individuals and communities, so while some people may identify with their use of drugs as part of their core identity, many may not. This means the term ‘community’ has to hold a multitude of different meanings, which are all highly dependent on context. With so many shifting definitions and different ways of understanding and communicating about ideas, identity and belonging, agreeing on who should or can represent such a large, dynamic and diverse group of people can be a contentious topic. While there may be a kind of objective criteria (in our case, using drugs), the boundaries of identity for people who use drugs are porous and authenticity may be contested. At any rate, it is not possible or feasible to engage everyone or most people who inject drugs, and we are attentive to the limits of representation.

There is limited funding available, and so it can be challenging, or even impossible for drug user organisations to ensure they are addressing the primary concerns of this diverse and multi‐faceted population. It would be impossible to engage any and every group who could possibly be associated with, or identified as someone who uses drugs. For example, there are over 3.9 million people who use drugs in Australia and AIVL receives less than $700,000 per year in federal funding available across the entire country allowing the employment of less than 10 employees with lived‐living experience.

Being mindful of the limits of representation and desiring to be inclusive of a diversity of voices and opinions of people who use drugs, AIVL, as the national peak body undertook a consultative process early in the grant idea phase. An open consultation was held with consumers/community members about the research agenda, to inform the research plans for the grant. The focus of the consultation was on research priorities. Yet there is a gap between what might be considered a research priority and what might be an immediate health need. For many people who use illicit drugs, there is a suffocating wall of unmet need amidst a vacuum of support, such that even necessities like food and basic hygiene for some people are not met. Asking about research priorities in this context feels empty and hollow. Conducting research is a slow, bureaucratic process. The low tolerance for risk and error in health research is at odds with the substantial and urgent health needs that are not being addressed for people who use drugs. There is a further issue with moving from identifying a priority health outcome or policy change versus what is ‘researchable’ and fits within a research grant application and associated program of work.

Despite the commitment to an open consultation process, the existing power imbalance between community partners who are directly impacted by the issues being researched (in our work, this includes individuals who work at a DUO, and also directly impacted individuals who work within the university) and researchers who are not living the issues being researched may impact participation. Being a researcher is a socially valued position [22]. Being someone who uses drugs is a highly devalued and stigmatised position. In this context, pressure to centre and regurgitate the views and desires of people with more valued positionalities on the team may be intense. Internalised stigma [23] and recognition of being in a context with which they are not familiar may also contribute to self‐silencing for community members. Alternately, those in positions where they are used to being valued, in the context of an environment in which they are familiar, the research environment, may result in researchers having difficulty prioritising the voices of people they have often worked with as ‘subjects’ of their research rather than equals.

Consulting on study design and research priorities for people who are not familiar with research can be challenging. For individuals who use drugs not employed in DUOs or research institutes, and even for many people employed in DUOs whose roles don't involve research, it would be unreasonable to expect them to conceptualise and articulate problems they encounter in ways that are readily translated and understood as being research priorities. In addition, skills in leading consultations are necessary. Building capacity for consumer representatives to facilitate consultations on research priorities is much bigger than any single grant application. The same is true of researchers—who do not innately have consultation skills. For both researchers and community representatives it additionally requires a commitment to working with an ethic of care to build enough trust and support to effectively help people who may come from marginalised backgrounds to openly share insights. Being able to get those insights out of people, takes time, money and skills.

For individuals who use drugs, and who are either researchers at universities or work within DUOs, they must navigate these tensions between the broader community of people who use drugs, and their relatively privileged status within it, the lack of resourcing and capacity, and also ensure that they do not violate the unwritten terms of representation imposed on them by other professionals with lived‐living experiences [24]. They must also simultaneously navigate the expectations of people they work with who do not have lived‐living experience of drug use, many of whom may be uncomfortable or unfamiliar with issues related to drug use and drug culture, or some may even be afraid of them [25].

Consumers experience other stresses as collaborators, notably in their role as ‘representatives’ with the attendant worry as to how individual behaviour may reflect on the ‘community’. Lived‐living experience of criminalised drug use holds limited social capital only in some very specific, limited contexts. The incongruity of simultaneously having this lived‐living experience conditionally valued, while it remains criminalised and highly stigmatised can be painfully hard to navigate. The pressure to perform as if you are not impacted by the issues for which you are valued may come from all directions. Essentially, consumer representatives can feel as if they have been told, ‘You/we are here because you/we are directly impacted by an issue of interest to research, but the expectation is that one should never seem like they are directly impacted by this issue’.

### 3.4 Sharing Power With, Not Power Over: Community Partners or Community‐Led Research?

When done well, research partnerships between community and university researchers can have uncomfortable moments. One example was the team member with living experience of injection drug use employing visual representations of meeting notes (including pink glitter, flowery lettering and graphic images of drugs) which one of the researchers found alienating, unable to marry the meeting content as they understood it to the pictorial summary—a useful turnaround of power. It can be uncomfortable to share power, and it begs the question—do researchers see themselves as equal to the highly stigmatised people they have spent years treating as ‘subjects’ of their research? It does come down to a question of values; can researchers challenge societal norms, overcome their position of power and adopt a set of values that uphold socially devalued individuals as equal partners? Recognising that the criminalisation of drug use casts a chilling shadow, limiting safe engagement in research, can people who use drugs take power while facing this perpetual threat, and being punished and criticised for holding divergent or even opposing views to those held by researchers or by other community members? Some people who use or inject drugs may find that being exposed to unkindness and disrespect over several years or even decades, has hurt their ability to advocate effectively [26]. For some community members, the experience of stigma in their current interactions with academic researchers is ongoing and has created an expectation of chronic under‐resourcing. Some academic researchers continue to expect that community members make their expertise available to benefit their work, and their contributions might be reimbursed (if they are reimbursed at all) with gift cards of a nominal amount. For anyone who partners with community to conduct research, or who would like to engage in community research, it is important to be mindful of the experience partners have had in the past.

Inherent to the research funding process is the decision about the fund‐holder (or auspice organisation). In the case of many research funds, it must be an academic institution (as is the case with NHMRC). We noted in our discussions that the grant, by default, needs to be held by an institutional/academic partner. What effects does this bring in terms of power‐sharing? Deliberations about the distribution of the funding (should a grant be successful) include considerations regarding how much funds are assigned to the community partners versus to the academics. From the outset, we approached this collaboration as one of true power sharing, with for example, the ideal of equal numbers of Chief Investigators from the community as from academia and shared decision making about all relevant aspects of the project. Indeed, our original language was a ‘community‐led’ project, reflecting our aspirations and commitments to community leadership. Over time and as further discussions amongst collaborators were held, it became apparent that it could not be ‘community‐led’ within the current NHMRC framework. Most significantly, the system within which we were operating (in this case the NHMRC guidelines for the funding opportunity) was not designed to genuinely accommodate a ‘community‐led’ grant application where the grant would be controlled by a majority of academics.

While our community partners bring decades of experience and expertise to our project, none of them possess the research track record required by funders to make a competitive grant application. Additionally, the university has many resources that a small, community organisation does not have and which would be very challenging for a small organisation to establish if a grant application is successful. In addition to issues of data ownership and sovereignty, tasks like data ownership, protecting and storing any data collected during research, would likely be burdensome and expensive for our community partners to take over and lead. Researchers have also spent a lot of time learning how to develop research projects, honing research skills and leading research projects and while these skills can be taught to others who have not had the same educational or professional research experience, many of these skills are not useful for the work the community members usually undertake and may hold very little value for our community partners. The time and resourcing it would take to both teach and learn these skills is also not funded or easily available within the development of a research grant proposal. There are aspects of research that consumers may experience as burdensome and, understandably, they may have no interest or desire to take on additional work that they may not see any benefit from. It would be unproductive to try to push for them to do so merely to fulfil some goal of restructuring hierarchy. It was at this point of recognition and acknowledgement of the system within which we were working, and the realities of working together, that our language shifted to ‘partnership’. In reflecting on this evolution from ‘community‐led’ to ‘consumer‐researcher partnership’, we were forced to recognise that it would be disingenuous to call something ‘community‐led’ when the lead CI was an academic, funding and any data we collected over the course of our research would be held by a research institute and the majority of CIs would be researchers. On the other hand, for our community partners, losing the opportunity to lead, even if it was never real and continuing this work in a situation of disempowerment as the new base moving forward felt like a loss and impacted engagement for a community that has lost so much already. It also opened us to the reality of research practices: there are specific and specialised skills associated with applying for research grants that are not necessarily held by consumers. Further, there are specialised expertise and skills held by consumers and community organisations which are typically not within researchers' portfolios. Recognising that specialist skills are not evenly distributed and valuing each equally is one of the first steps to ensure we are sharing power and working to collaborate equitably.

## 4 Conclusions

Our attempt at making a grant application as a truly equitable partnership between researchers and consumers reveals the tension between the rhetoric of meaningful consumer and community involvement promoted by funding bodies like the NHMRC, and the practical realities of achieving equitable partnerships in research. We would like to note that although we have used this experience with applying for an NHMRC grant as an example, our experience with other funding bodies is similar. In fact, the NHMRC has made incredible progress over the past decade to ensure the meaningful involvement of consumers in research and we are hoping to provide useful information to continue supporting this important evolution. It is simply that some of the systemic barriers such as the grant application documentation, grant application requirements and peer review guidelines have not completely caught up with these well‐meaning efforts.

While many of the documents and resources provided by funders consistently emphasise co‐production and shared power, the inherent power imbalances within academic structures and funding processes create significant barriers to achieving this. Grant application criteria, success metrics and even the language used, often prioritise traditional academic achievements over genuine community engagement. This inadvertently devalues and marginalises community members' lived‐living experiences and expertise, hindering authentic and meaningful participation and potentially compromising the relevance and impact of the research.

There are several ways in which the gap between rhetoric and practice can be bridged. Researchers must invest more in building trust, providing adequate resources for meaningful community and consumer organisation involvement and adopting flexible, culturally appropriate methods of collaboration that genuinely centre community perspectives. Understanding and appreciating power imbalance, and also the complex issues associated with representation (who is representing the consumers) need to be ongoing active discussions. Institutions can address the structural and financial barriers that researchers may face, and actively support and facilitate meaningful consumer engagement across the spectrum of research development, grant writing and research conduct. Finally, funding bodies need to reconsider the success metrics by which grant applications are judged, ensuring they accurately reflect equitable partnerships and reassess grant application processes to remove systemic biases that disadvantage community‐led approaches. Funders need to align their actions with their commitment to consumer engagement in research. Only through such fundamental shifts in research practices, institutions and funding bodies can we ensure that research is truly relevant, impactful and ethically sound and has an impact for the communities it is meant to serve.

## Author Contributions

Conceptualization: All authors; Supervision: Jason Grebely, Alison Ritter; Writing – Original Draft Preparation – Danielle M. Russell, Ele Morrison, Jason Grebely, and Alison Ritter; Writing – Review and Editing – All authors.

## Funding

The Kirby Institute, UNSW Sydney, is funded by the Australian Government Department of Health and Aged Care. The views expressed in this publication do not necessarily represent the position of the Australian Government. J.G. is supported through National Health and Medical Research Council (NHMRC) Investigator Grants (1176131, 2034002). K.S. holds an Australian Research Council (ARC) Future Fellowship (FT200100099). A.R. holds an NHMRC Investigator Grant (2016695).

## Conflicts of Interest

C.T. has received speaker fees from Gilead Sciences. J.G. is a consultant/advisor and has received research grants from Abbott, AbbVie, bioLytical, Cepheid, Gilead Sciences, Hologic and Roche. D.M.R. has received speaker fees from Gilead Sciences.

## Data Availability

The data that support the findings of this study are available from the corresponding author upon reasonable request.
